# How Tool-Use Shapes Body Metric Representation: Evidence From Motor Training With and Without Robotic Assistance

**DOI:** 10.3389/fnhum.2019.00299

**Published:** 2019-09-12

**Authors:** Valentina Bruno, Ilaria Carpinella, Marco Rabuffetti, Lorenzo De Giuli, Corrado Sinigaglia, Francesca Garbarini, Maurizio Ferrarin

**Affiliations:** ^1^MANIBUS Laboratory, Psychology Department, University of Turin, Turin, Italy; ^2^IRCCS Fondazione Don Carlo Gnocchi, Biomedical Technology Department, Milan, Italy; ^3^PHI-LAB, Department of Philosophy, University of Milan, Milan, Italy

**Keywords:** coexistence between goal representation and bodily movements, peripersonal space, tool-use, body metric representation, robotic assistance, passive movements

## Abstract

Previous evidence has shown that tool-use can reshape one’s own body schema, extending peripersonal space and modulating the representation of related body parts. Here, we investigated the role of tool action in shaping the body metric representation, by contrasting two different views. According to a first view, the shaping would rely on the mere execution of tool action, while the second view suggests that the shaping induced by tool action on body representation would primarily depend on the representation of the action goals to be accomplished. To this aim, we contrasted a condition in which participants voluntarily accomplish the movement by representing the program and goal of a tool action (i.e., active tool-use training) with a condition in which the tool-use training was produced without any prior goal representation (i.e., passive tool-use training by means of robotic assistance). If the body metric representation primarily depends on the coexistence between goal representation and bodily movements, we would expect an increase of the perceived forearm length in the post- with respect to the pre-training phase after the active training phase only. Healthy participants were asked to estimate the midpoint of their right forearm before and after 20 min of tool-use training. In the active condition, subjects performed “enfold-and-push” movements using a rake to prolong their arm. In the passive condition, subjects were asked to be completely relaxed while the movements were performed with robotic assistance. Results showed a significant increase in the perceived arm length in the post- with respect to the pre-training phase only in the active task. Interestingly, only in the post-training phase, a significant difference was found between active and passive conditions, with a higher perceived arm length in the former than in the latter. From a theoretical perspective, these findings suggest that tool-use may shape body metric representation only when action programs are motorically represented and not merely produced. From a clinical perspective, these results support the use of robots for the rehabilitation of brain-damaged hemiplegic patients, provided that robot assistance during the exercises is present only “as-needed” and that patients’ motor representation is actively involved.

## Introduction

Acting with tools is a familiar aspect of everyday life. People use tools for eating cakes, moving logs, picking up leaves, and writing papers. A characterizing feature of tools is that they often make out-of-reach objects reachable and manipulable. There is a lot of evidence that using a rake-like tool exerts a deep impact on the agent’s space representation, enlarging her own reaching space according to the range of tool action. It has been demonstrated that tools are treated by the nervous system as sensory extensions of the body rather than as simple distal links between the hand and the environment (Miller et al., 2018). A seminal study by Iriki et al. (1996) on non-human primates showed that a repeatedly used small rake expanded the receptive fields of parietal visuo-tactile neurons to encompass the space around both the hand and the rake. If the monkey held the rake without using it, the receptive fields shrank back to their usual extension. Analogous results have been obtained in both healthy and brain-damaged humans. For instance, studies on healthy subjects showed that tool-use might increase the impact of far visual distracters on tactile discrimination (Maravita et al., 2002; Holmes et al., 2004) as well as the sensitivity to the affording features of out-of-reach objects (Costantini et al., 2011). Similarly, studies on patients with visuo-tactile extinction indicated that the severity of their extinction could be modified by using tools, which extend the reach of hand actions (Farnè and Làdavas, 2000; Maravita et al., 2001; Farnè et al., 2005). In the same vein, patients with neglect only for the hemispace close to their body have been found to worsen their performance in a line bisection task in the far space when using a tool like a long stick (Berti and Frassinetti, 2000; Neppi-Mòdona et al., 2007).

Strikingly, tool-use has also been reported to affect the agent’s body representation (Martel et al., 2016). For instance, it has been shown that tool-use might alter the kinematic profile of forearm movements in a reach to grasp task. Even more interestingly, tool-use has been found to modify body metric representation (Cardinali et al., 2009a). Sposito et al. (2010) took advantage of an arm bisection paradigm (Bolognini et al., 2012; Tosi et al., 2018), by asking participants to estimate the subjective midpoint of their own forearm before and after a training phase with long (60 cm) and small (20 cm) tools (Sposito et al., 2012). The results showed that participants indicated a more distal midpoint, thus exhibiting an increased representation of the length of the arm handling the tool, after long-tool-use training only. Indeed, using small tools did not alter participants’ body metric representation. More recently, Romano et al. (2019) have investigated how different actions with a tool may impact the subjective metric representation of the body. They found a proximal shift in the perceived midpoint when the training phase with tool mostly involved proximal movements (e.g., shoulders), while a distal shift occurred after the training phase asking for a large use of proximal movements (wrist and fingers).

There is a mounting consensus that the representation of the body is similar to the representation of the surrounding space with respect to its being action-oriented (Maravita and Iriki, 2004). In a nutshell, this means that body representation is not only sensory but also motor in nature, and it is for this reason that actions may shape how the body is represented (Gallese and Sinigaglia, 2010). Acting with tools makes this point vivid. As the aforementioned studies indicate, tool actions can alter agents’ body metric representation, with this effect being related both to which tool is used and how it is used. However, postulating a link between body and action allows two different and (partially atleast) alternative views on how tool actions may shape the way in which the body is represented.

According to a first view, the shaping would rely on the mere motor execution of tool actions. Some evidence speaks for this first view, albeit indirectly. For instance, it has been shown that tools have to be effectively used to reach far objects, since just holding them (Iriki et al., 1996; Farnè and Làdavas, 2000; Maravita et al., 2001; Serino et al., 2007) is not enough to alter space representation (Serino, 2019). It seems therefore natural to assume that something similar holds for body representation. But this assumption could be disputed by a second view, according to which the possibility for tool action to shape body representation would primarily depend on the coexistence between goal representation and bodily movements. According to this view, in order for the tool-use to shape the body representation, goals and motor programs have to be represented to intentionally accomplish tool actions. There is some evidence supporting this second view. For instance, it has been shown that imaging acting with tools is sufficient to modify one’s own arm’s length representation (Baccarini et al., 2014). Furthermore, Garbarini et al. (2015a) reported the case of brain-damaged hemiplegic patients who manifested a pathological embodiment of other people body parts (Fossataro et al., 2016, 2018b; Ronga et al., 2019). The patients were asked to estimate the midpoint of their paralyzed forearm before and after a training phase in which an experimenter repeatedly used a tool, being aligned or misaligned relative to patients’ shoulders. When the experimenter was aligned, the patients were (delusionally) believing to perform the tool-use training with their own paralyzed arm. This induced a significant modulation of the perceived arm length. Indeed, the patients located their forearm midpoint more distally (i.e., close to the hand) in the post- than in the pre-training phase. No effect occurred when they were misaligned to the experimenter during the training phase (Garbarini et al., 2015a). Other evidence supporting the second view comes from two studies of Cardinali and colleagues in healthy subjects. In a first study (Cardinali et al., 2009b), when investigating the differential role played by the morpho-functional characteristics of a tool and the sensorimotor constraints that a tool imposes on the hand, they found that tool-use induces a rapid update of the hand representation in the brain, not only on the basis of the morpho-functional characteristics of the tool but also depending on the specific sensorimotor constraints that each tool imposes to the user’s motor program. In a second study (Cardinali et al., 2012), when assessing functional against non-functional tool-use with respect to the effects on body representations, they found that the same tool, used for different tasks (i.e., a grabber to grasp object or a grabber to perform a perceptual task), differently affects arm length representation, depending on how it is used. This suggests that our perceived body metrics is differently modulated, according to the way in which specific goals and motor programs of a tool action are represented.

The main aim of the present study is to specifically investigate how tool action may shape body representation, by contrasting these two views. In doing this, we need a pair of situations that differ in that one involves the representation of the tool action goals and motor programs, whereas the other does not. To create such a pair of situations, we adapted the arm bisection paradigm used by Sposito et al. (2012) and Garbarini et al. (2015a), by contrasting a condition in which there is a coexistence between action goals to be accomplished and bodily movements (i.e., active tool-use training) with a condition in which the tool-use training was produced without representing a corresponding action goal (i.e., passive tool-use training by means of robotic assistance). The comparison between active and passive movements has been previously used to dissociate the representational component of the movement from the mere displacement of our body in space, by using different techniques such as hand-twitches induced by single-pulse transcranial magnetic stimulation (e.g., Bolognini et al., 2016; Bruno et al., 2017) and limb mobilization induced by mechanical device (e.g., Bisio et al., 2017; Fossataro et al., 2018a) and by the experimenter during ischemic nerve block (Christensen et al., 2007) or during resting condition (Garbarini et al., 2015b). Upper limb movements have been studied in healthy people and subjects with neurological conditions also by taking advantage of robotic arms, since they are able to produce different force fields aimed at enhancing the subject’s residual motor control or at imposing highly controlled, reliable, and repeatable passive movements (Patton and Mussa-Ivaldi, 2004; Carpinella et al., 2009, 2012; Pan et al., 2011; Casadio et al., 2015; Cardis et al., 2018). Irrespective of the techniques employed, the common feature of the passive movement is the lack of the intentional component and, therefore, the consequent absence of motor representation. Indeed, during passive movements, subjects do not have to represent the goal of the action in order to voluntarily produce it, but their actions only depend on externally generated forces.

If tool actions may shape the body representation by virtue of their effective production (first view), no differences in the subjective metric estimation of the body after active and passive training should be expected. On the contrary, if the body metric representation primarily depends on whether, during tool-use, the action programs and goals are motorically represented rather than merely produced (second view), we would expect to find a significant increase of the perceived forearm length in the post- with respect to the pre-training phase after the active training phase only.

## Materials and Methods

### Participants

Twelve healthy participants (five females; mean age ± SD: 24.3 ± 1.4) took part in the study. The sample size was based on our previous study exploring the modulation of the right arm body metric representation after tool-use training (i.e., *N* = 10; in Garbarini et al., 2015a). A similar sample (*N* = 11) was used in the original article of Sposito et al. (2012). Therefore, in the present study, 12 participants were recruited in order to obtain a sample of at least 10 participants showing the modulation of the right arm body metric representation after tool-use training (see details in “Experimental Paradigm” section). All participants were right-handed (Oldfield, 1971) and naïve to the purpose of the experiment. None of them had history or evidence of neurological, psychiatric, or other relevant medical problems. Participants gave informed written consent. The study was approved by the Ethics Committee of the Don Carlo Gnocchi Foundation IRCCS (session 2014-12-10) and conforms to the Declaration of Helsinki.

### Experimental Paradigm

**Figure 1:**
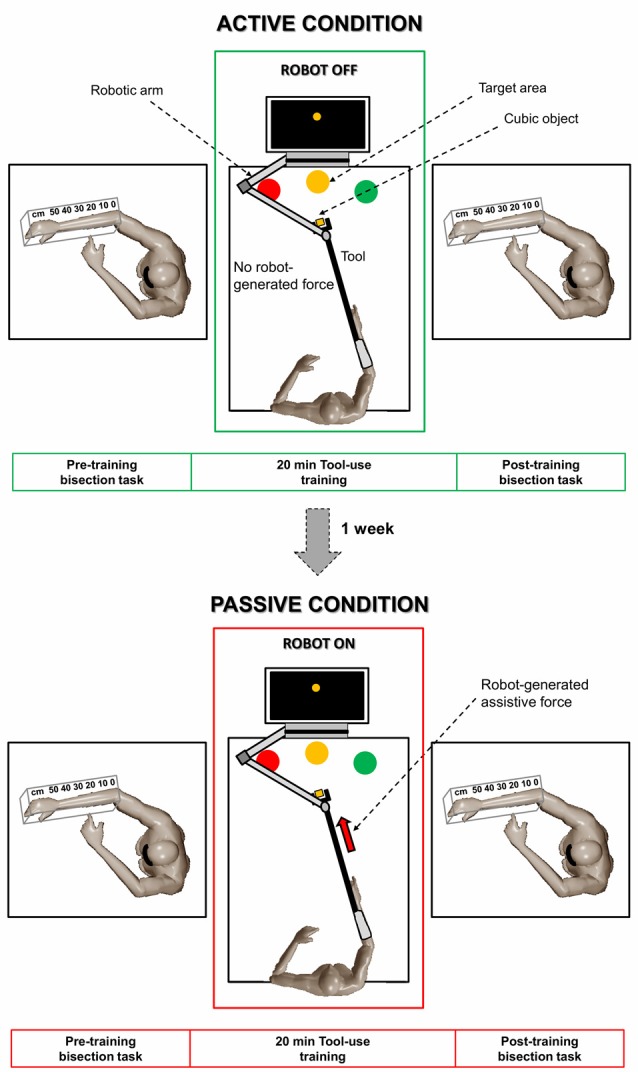
Schematic overview of the experimental paradigm. Each participant performed the forearm bisection task immediately before and after 20 min of tool-use training in two different sessions, separated by a week: active session (upper part) and passive session (lower part). The tool-use training consisted of an “enfold-and-push” task. In the active session, the robot did not provide any force and the subjects actively performed the movements. In the passive session, the robot generated an assistive force that moved the tool (and consequently the forearm) towards the target area.

**Figure 2:**
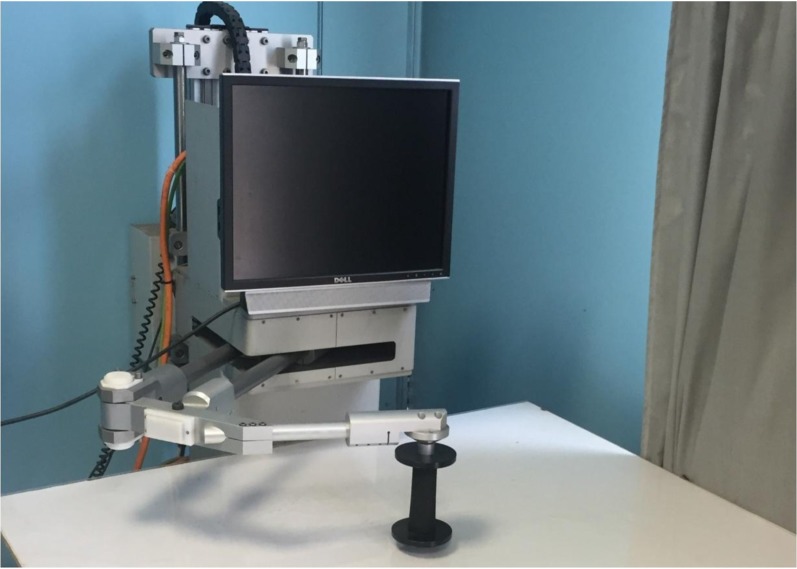
Picture of the robot used in the present study.

The experimental paradigm is shown in Figure 1. Participants performed a forearm bisection task (for more details, see the next section) immediately before and after 20 min of tool-use training. The tool-use training was performed by means of the planar robot for the upper limb shown in Figure 2 (Braccio di Ferro, Celin, Italy; Casadio et al., 2006), which was equipped with a customized handle. The handle connected the robotic arm to a tool consisting of a 120-cm wooden rod with a U-shape extremity (i.e., the rake). The opposite extremity of the tool was fixed to participants’ right forearm through a bondage to prolong their arm. After preparation, participants, sitting in a comfortable position with both forearms on a table, underwent the tool-use training involving the repeated execution of “enfold-and-push” movements. In particular, for each repetition, one of three cubic objects (green, yellow, and red cubes with a side of 3.5 cm) was placed on the table by the operator in random order at a distance of 120 cm from anterior torso along participants’ midsagittal plane. Therefore, the object had to be “enfolded” by the participants using the U-shaped extremity of the tool and smoothly pushed to the target area with the same color as the moved cube (see Figure 1). This robotic version of motor training is functionally similar to the “grasp-and-place” task previously employed in previous studies (Garbarini et al., 2015a; Romano et al., 2019). The three target areas were placed at a distance of 20 cm from the starting position respectively at 60°, 90°, and 120° from the horizontal to cover a significant part of the reaching space. Each participant performed the tool-use training in two different sessions, separated by a week: active and passive. In both sessions, participants were asked to execute the “enfold-and-push” task for 20 min. In the active session, the robot did not provide any force toward the target area and the subjects actively performed the movements. During the passive session, performed after a week, the robot generated an assistive force that moved the tool (and consequently the forearm) towards the target area. The assistive force was implemented *ad hoc* in order to impose to the robotic handle a minimum-jerk trajectory that is typical of reaching movements naturally executed by healthy subjects in real-life contexts (Flash and Hogan, 1985). In the passive session, the participants were asked to relax as much as possible and to let the robot move their arm without any active intervention. Both the active and passive training sessions were performed with the eyes open. In the passive session, only participants (*N* = 10, 5 females; mean age ± SD: 24.4 ± 1.2, according to the sample size of the previous mentioned studies) showing the classical pattern of modulation of the perceived arm length after the active session (see details in “Forearm Bisection Task” section) were called back; therefore, the active session was always performed first. Previous evidence with this paradigm showed no sequence effect in the active condition if performed in two different sessions at 1 week of distance (Garbarini et al., 2015a); therefore, it makes it unlikely that any difference found between the two manipulations in the present study (active and passive) should be due to the sequence order (active first).

### Forearm Bisection Task

The experimental task consisted in a forearm bisection task already used in previous studies aiming at investigating the effectiveness of tool-use training (e.g., Sposito et al., 2012; Garbarini et al., 2015a). While blindfolded, participants were instructed to indicate, by using their left index finger, the midpoint of their right distal upper limb segment comprising the forearm and the hand, considering the elbow and the tip of the middle finger as the two extremities. During the task, in order to prevent any possible tactile feedback from the bisections, the right forearm was kept in a radial posture and placed inside a Plexiglas parallelepiped (70 × 10 × 11 cm^3^). On the top of the Plexiglas screen, above the arm, a paper ruler with centimeters was attached. The 0 cm of the ruler corresponded to the elbow, in order to easily measure the position of the subjective midpoint (*p*). Then, in order to obtain a percentage score relative to each participant’s subjective arm length, we used the following formula: [(*p*/arm length in cm) × 100]. During the task, corrections were not allowed. In each session (i.e., active; passive), each participant performed a total of 30 forearm bisection judgments, 15 before (pre-training) and 15 after tool-use training (post-training; Sposito et al., 2012; Garbarini et al., 2015a).

### Statistical Analysis

The mean forearm bisection value obtained for each subject in each session (i.e., active; passive) before and after the tool-use training was used as the dependent variable. These data were entered in a 2 × 2 repeated-measures ANOVA with two within-subject factors Session (two levels: active; passive) and Time (two levels: pre-training; post-training). *Post hoc* comparisons were performed by means of Newman–Keuls test. The analysis was performed using Statistica software 8.0 (StatSoft, Inc., Tulsa, OK, USA). We reported mean, standard deviation, and *p*-value, and when a significant effect was found, the effect size (*η*^2^) and power were reported as well.

## Results

**Figure 3:**
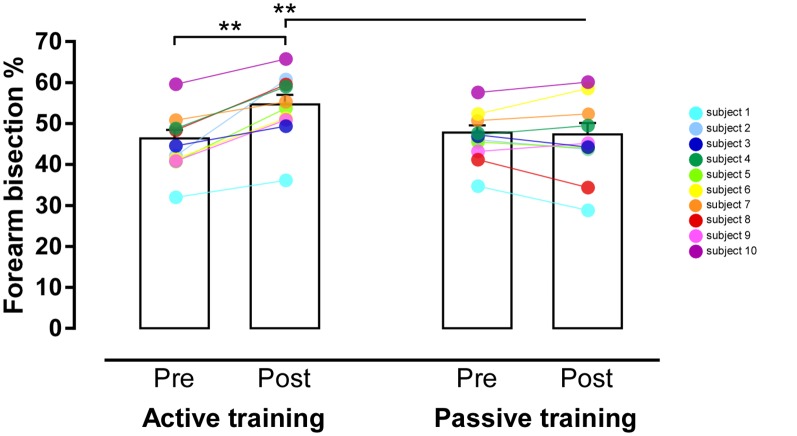
Results of the Task*Time interaction. Graphic representation of themean forearm bisection values (in %) in participants performing theactive tool-use training (on the left) or the passive tool-usetraining (on the right) in the pre- and post-training conditions. Theeffect of training is significant only in the active condition; no difference between pre- and post-training was found in the passive condition. Error bars represent standard error of the mean. **p < 0.001.

With respect to the mean forearm bisection values, the ANOVA found a significant main effect of Time (*F*_(1,9)_ = 25.47, *p* = 0.0007, *η*^2^ = 0.74, power = 0.99), with significantly greater values (i.e., increased arm length perception) in the post-training than in the pre-training phase. Crucially, a significant Session*Time interaction (*F*_(1,9)_ = 21.04, *p* = 0.001, *η*^2^ = 0.7, power = 0.98) was found, suggesting that the perceived length of the forearm was modulated by the session. In particular, *post hoc* comparison showed a significant increase of the perceived arm length in the post- with respect to the pre-training phase in the active session (*p* = 0.001; Figure 3), while no difference emerged between the post- and the pre-training phase of the passive session (*p* = 0.76). It is important to note that the pre-training of both the active and passive session did not differ (*p* = 0.08), but interestingly, the post-training phases of both sessions were significantly different (*p* = 0.008), with a significant increase of the perceived arm length in the post-training of the active with respect to the post-training of the passive session. Furthermore, the post-training phase of the active session was significantly different from all the other conditions (*p* always <0.01 for each comparison; percentage score relative to each participant’s subjective arm length, mean ± SD: pre-training active = 46.4 ± 6.7; post-training active = 54.7 ± 7.3; pre-training passive = 47.8 ± 5.6; post-training passive = 47.4 ± 8.7).

## Discussion

The present study aimed at investigating how tool-use may shape the body metric representation. We contrasted two different and (partially at least) alternative views. According to the first view, the actual execution of tool action would be enough for the shaping to occur, while the second view postulates that a coexistence between action goals to be accomplished and bodily movements is necessary; i.e., it is not enough that the bodily movements are merely executed, the action programs and goals have to be motorically represented. Body metric representation was measured by means of a forearm bisection task. In this task, participants were asked to indicate the midpoint of their right upper limb segment comprising the forearm and the hand, considering the elbow and the tip of the middle finger as the two extremities (Sposito et al., 2012). The forearm bisection task was performed before and after two different tool-use training sessions. Indeed, participants underwent a session in which they *actively* performed 20 min of tool-use training and a session in which the tool-use training was *passively* performed by means of robotic assistance. The main finding was that participants exhibited a significantly increased arm length estimation in the post- with respect to the pre-training phase after the active session only. Indeed, when the tool-use training was performed in the Passive session, in which participants were instructed to maintain a relaxed posture while the robot passively moved their arm, no modulation of the perceived arm length occurred. This suggests that the mere production of tool action is not enough for shaping agent’s body representation. Specific motor programs of the tool action need instead to be voluntarily implemented and represented.

Our finding is in line with some previous studies suggesting a role of motor processes and representations in the subjective estimation of body metric. For instance, Garbarini et al. (2015a) showed that hemiplegic patients may increase the length estimation of their paralyzed forearm after a training phase in which an experimenter was aligned to them and repeatedly used a tool. Indeed, the patients showed a pathological embodiment of the experimenter’s arm, thus having real intentions to move the tool as if they were actually performing the training with their own paralyzed arm. And this was enough for the perceived arm length increase to occur, or so the authors argued. In a similar vein, a very recent study on healthy subjects has demonstrated that body metric estimation can be modulated by the sense of agency (D’Angelo et al., 2018). Participants were asked to perform a forearm bisection task before and after a training phase, in which they virtually grasped objects and make precision grip by controlling a far 3D virtual hand. The training phases consisted of two conditions characterized by a different timing in the visual feedback. In a synchronous condition, participants were shown virtual hand movements responding in real time to their own right-hand movements, while in an asynchronous condition, a 3-s delay was interposed between the participants’ real hand and the virtual hand movements. The results showed that participants pointed to their forearm midpoint more distally after performing the training phase in the synchronous condition, where they sensed agency for the far virtual hand. According to their results, only if participants sensed agency for the virtual hand, induced by the synchronicity, and therefore experienced a sense of congruency between the intention to perform the action and the motor output coming from the movement performed did they show the classical modulation of body metrics. Similarly, the notion of congruency is ubiquitous within the body literature. We experience the rubber-hand illusion (Botvinick and Cohen, 1998) under the synchronous condition but not under the asynchronous condition, for instance. More specifically, in the context of tool-use, it has been demonstrated that the peri-personal space expands after a session of near touch and congruent visual stimuli presented far (Serino et al., 2015). This is not the case when training was incongruent. Accordingly, in our study, the shaping of the body metric representation occurs only when there is a congruency between action goals and bodily movements, as in the active training.

Taken together, these and our findings indicate that motor processes and representations, involved in planning and monitoring tool action, may also play a critical role in shaping one’s own body metric representation. But how to explain this? A candidate hypothesis is that subjective estimation of body metric hinges on processes and representations which are not only sensory but also motor in nature. Planning and monitoring a tool action requires the agent to represent motorically both bodily and tool movements as if the tool was a part of the agent’s body (Gallese and Sinigaglia, 2010). This would involve not only an increase of the range of action, by making reachable things otherwise unreachable, but also a functional extension of the body, with the tool being incorporated much like a prosthetic device (Serino et al., 2007). Such incorporation does not occur if tool action is passively performed with the assistance of a robotic arm. There is here no need for an agent to represent her own body and action goals because tool action execution is fully driven by the robotic arm.

This hypothesis seems to be supported by evidence coming from different domains. For instance, Anelli et al. (2015) reported a similar dissociation between active and passive tool action in the time domain. Participants were asked to perform a time bisection task, by reproducing half of the duration of visual stimuli presented in near and far space, before and after an active tool-use training phase. The results showed a clear dissociation in the perceived duration between far and near stimuli. Indeed, participants exhibited a leftward bias in the time bisection task with near stimuli and a rightward bias with far stimuli. Strikingly, this dissociation disappeared after the training phase, since the far stimuli were perceived as nearer. In line with our findings, the dissociation did not disappear if the tool actions involved in the training phase were passively executed, without any motor preparation and control.

Similar results have been found in the spatial domain. There is a huge amount of evidence that tool actions may extend the agent’s space representation, with this extension occurring after short-term (Serino et al., 2007) as well as long-term (Serino et al., 2007; Bassolino et al., 2010) tool-use, even if the interpretation of the consequences of tool-use in the spatial domain is controversial (Holmes et al., 2004). Several studies took advantage of a cross-modal congruency task (Spence et al., 2004). In this task, participants speeded their performance when stimuli from different modalities (e.g., tactile and visual) are temporally and spatially congruent. Indeed, it has been shown that the detection of tactile stimuli delivered to the body is more effectively influenced by visual (Macaluso and Maravita, 2010) or auditory (Occelli et al., 2011) stimuli occurring near to, as compared to far from, the body. Interestingly, short-term tool-use has been found in healthy subjects to increase the impact of far visual distracters on tactile discrimination (Maravita et al., 2002; Holmes et al., 2004). Analogously, acting with a tool, which gets things otherwise out-of-reach, has been demonstrated in brain-damaged patients to expand visuo-tactile extinction from near to far space (Farnè and Làdavas, 2000; Maravita et al., 2001; Farnè et al., 2005). As far as long-term tool-use is concerned, blind cane users provide a paradigmatic case of extensive and functionally highly relevant population that constantly perform actions by means of a tool. Serino et al. (2007) asked blind cane users and sighted subjects to respond as soon as possible to tactile stimuli on their hand, while ignoring concurrent sounds presented either near to the stimulated hand or approximately 120 cm far from it, before and after a training phase, which consisted in exploring the far space with a cane. The results showed that sighted subjects responded faster to tactile stimuli associated with far sounds after the training phase only. The effect was absent before the training phase and disappeared when the sighted subjects no longer used the cane. On the contrary, holding the cane, without actually using it, was enough for the blind subjects to result in faster reaction times to touches coupled with sounds occurring at the far space (i.e., at the tip of the cane). Things were different when the blind subjects held a short handle. As in sighted subjects before the training phase, reaction times were faster to tactile stimuli associated with near sounds only (Serino et al., 2007).

All these results point to a change in the way in which the body and the space around it are represented when tool actions are planned and monitored, suggesting that these actions may involve a short or even a long-term tool embodiment, such that the tool becomes part of the acting body (Berti and Frassinetti, 2000; Maravita et al., 2001; Farnè et al., 2005). However, although body and space representations are strictly related, this does not imply that they both rely on the same processes and mechanisms. For instance, in the Galli et al. (2015) study, healthy subjects performed a training with a very special tool (i.e., wheelchair) in Active and Passive conditions and, after that, they underwent a classical audio-tactile looming task (Canzoneri et al., 2012; Serino et al., 2018) used to evaluate the post-training effect on the peripersonal space representation. They did not find the expected results after the active condition, likely because, as proposed by the authors, the very unfamiliar tool action (such has moving a wheelchair for healthy subjects) might have prevented the occurrence of the external space remapping, by shifting the attention on the internal motor effort. Interestingly, they found a remapping of the peripersonal space after a passive training (i.e., when the wheelchair was pushed by someone else) but only when participants can see the explored environment (and not when they are blindfolded). On the same vein, Costantini et al. (2011) have systematically investigated how tool action affects space representation. They found that not only actively using a tool but also merely observing someone else using a tool may extend one’s own reaching space. For the extension to occur, the observer had to do nothing more than holding a tool compatible with the goal and the spatial range of the observed action, thus sharing the same action potentialities with the observed agent. It makes sense that visual information, when present in Passive condition (Galli et al., 2015), as well as in observation condition (Costantini et al., 2011), plays a crucial role in shaping the coding of the space around the body. A different result was obtained when the effect of tool-use observation on body representation was investigated. Garbarini et al. (2015a) asked participants to perform a forearm bisection task after and before observing someone else performing tool actions. The results did not show any modulation of the perceived arm length, even when the participants held a tool compatible with the observed action. Although further research is needed, this indicates that, differently from space representation, the representation of the body is mostly sensitive to motor processes and representations typically involved in planning actions and monitoring their execution. Since here, as in the latter study, we focused on body representation (and not on space representation), it is likely that visual information, commonly available during both active and passive training, may result in a less effective shaping of the space representation, thus making unaffected our forearm bisection task.

To sum up, when there is a coexistence between action goals and bodily movements, tool-use may shape body metric representation. Otherwise said, whether people represent (or do not represent) the program and the goal of their actions, when using a tool, has important consequences on what they perceive about the length of their body parts. This can be of interest not only from a theoretical but also from a clinical point of view. First, the present findings confirmed that motor planning and control play a crucial role for the promotion of motor learning, which is responsible for the plastic changes in body representation (Classen et al., 1998; Benarroch, 2006) and is the basis of the rehabilitation in neurologically impaired subjects (Lotze et al., 2003). Indeed, if no active participation is provided, no motor learning is attained and, reasonably, no plastic modulation of body representation can occur, as found in the present study after the Passive condition. By contrast, it is well established that motor learning is promoted if the assistance is reduced to a minimum (assist-as-needed mode), allowing the subject to exert his/her residual voluntary control as much as possible during the execution of goal-directed movements (Sanguineti et al., 2009). This specific assistive mode, easily implementable in robotic devices, can therefore optimize the effect of rehabilitation through facilitation of motor learning and the promotion of neural plasticity.

## Data Availability

The datasets generated for this study are available on request to the corresponding author.

## Ethics Statement

The studies involving human participants were reviewed and approved by Ethics Committee of the Don Carlo Gnocchi Foundation IRCCS (session 2014-12-10). The patients/participants provided their written informed consent to participate in this study.

## Author Contributions

FG and CS conceived the study. FG, MR, IC, MF, CS, and LD designed the experiment. LD recruited the volunteers. IC implemented the robotic paradigm. LD and IC carried out the data collection. LD, VB, and MR analyzed the data. All authors participated in data interpretation. IC and VB prepared the figures. VB and CS drafted the manuscript. FG, MF, MR, and IC critically reviewed it. All authors approved the final version of the manuscript.

## Conflict of Interest Statement

The authors declare that the research was conducted in the absence of any commercial or financial relationships that could be construed as a potential conflict of interest. The handling Editor declared a shared affiliation, though no other collaboration, with several of the authors IC, MR, MF.
